# Activation of the Akt Survival Pathway Contributes to TRAIL Resistance in Cancer Cells

**DOI:** 10.1371/journal.pone.0010226

**Published:** 2010-04-19

**Authors:** Jing Xu, Jun-Ying Zhou, Wei-Zen Wei, Gen Sheng Wu

**Affiliations:** 1 Program in Molecular Biology & Genetics, Karmanos Cancer Institute, Department of Pathology, Wayne State University School of Medicine, Detroit, Michigan, United States of America; 2 Program in Breast Cancer, Karmanos Cancer Institute, Department of Immunology and Microbiology, Wayne State University School of Medicine, Detroit, Michigan, United States of America; University of Hong Kong, Hong Kong

## Abstract

The mechanism of tumor necrosis factor-related apoptosis-inducing ligand (TRAIL) resistance in cancer cells is not fully understood. Here, we show that the Akt survival pathway plays an important role in TRAIL resistance in human cancer cells. Specifically, we found that TRAIL treatment activates the Akt survival pathway and that inhibition of this pathway by the PI3K inhibitor LY294002 or knockdown of Akt sensitizes resistant cancer cells to TRAIL. Since Akt is negatively regulated by the tumor suppressor PTEN, we examined the TRAIL sensitivity in PTEN knockdown mouse prostate epithelial cells and found that PTEN^−/−^ cells are more resistant than PTEN^+/+^ cells while the sensitivity of PTEN^+/−^ cells fell in between. Further, we showed that overexpression of a mutant PTEN confers TRAIL resistance in PTEN^+/+^ cells, supporting a role of PTEN in TRAIL sensitivity. In TRAIL resistant breast T47D cells, overexpression of the mutant PTEN further increased their resistance to TRAIL. Taken together, our data indicate that inactivation of functional PTEN and the consequent activation of the Akt pathway prevents TRAIL-induced apoptosis, leading to TRAIL resistance. Therefore, our results suggest that TRAIL resistance can be overcome by targeting PTEN or the Akt survival pathway in cancer cells.

## Introduction

TRAIL (or Apo2L) is a member of the tumor necrosis factor superfamily that selectively induces apoptosis in cancer but not normal cells, making it an attractive agent for cancer therapy. TRAIL induces apoptosis through activation of its death receptors DR4 (TRAIL-R2) and/or DR5 (KILLER, TRAIL-R1, TRICK2) [1], [2], [3], [4]. When TRAIL binds to DR4 and/or DR5 receptors, the death receptors become trimerized and then recruit the adaptor protein FADD (Fas-associated protein with death domain) and procaspase 8 or procaspase 10 to form the DISC, leading to caspase 8 activation [1], [2]. Activated caspase 8 can directly activate the effector caspases 3, 6 and 7 to trigger apoptosis or indirectly induces apoptosis through the tBid-induced caspase 9 mediated mitochondrial apoptosis pathway [2], [4].

Although TRAIL is an attractive therapeutic agent many cancer cells are resistant to this agent [2]. The mechanisms of TRAIL resistance are not fully understood, but may occur at multiple levels from its receptors to executor caspases within its signaling pathway [2], [4]. At the receptor level, mutations in DR4 and DR5 have been found in some tumors including breast and lung cancers [4]. Increased expression of decoy receptors and decreased DR4/DR5 expression have been correlated with TRAIL resistance, although other studies indicated that the expression levels of TRAIL receptors do not correlate with TRAIL sensitivity. At the death-inducting signaling complex (DISC) level, c-FLIP is believed to be a major inhibitor for TRAIL signaling [5], [6]. A recent study showed that TRAIL sensitive non-small cell lung cancer (NSCLC) cells assemble DISC in lipid rafts of the plasma membrane that leads to caspase 8 activation, whereas non-raft DISC assembly leads to the recruitment of c-FLIP and RIP and the activation of the pro-survival signaling pathways such as NF-κB and ERK1/2 pathways [7]. In addition, it has been shown that anti-apoptotic members of the Bcl-2 family, including Bcl-2 and Mcl-1, are also involved in TRAIL resistance [8], [9]. The activation of other survival pathways including the Akt and NF-κB pathways leads to cell survival and chemoresistance [10]. Consistently, it has been shown that the activation of the Akt pathway is closely associated with drug resistance including TRAIL resistance [11], [12]. However, the exact mechanism by which the Akt pathway causes TRAIL resistance is not fully understood.

The PI3K/Akt signaling pathway is a prototypic survival pathway that plays a central role in diverse cellular functions, including proliferation, growth, survival, and metabolism [13]. Upon growth factor stimulation, PI3K is activated to phosphorylate phosphatidylinositol-3, 4-bisphosphate (PIP2), generating phosphatidylinsitol-3, 4, 5-triphosphate (PIP3). PIP3 binds to Akt, then translocates to the plasma membrane where Akt is activated by sequential phosphorylation at T308 and S473 residues. Phosphorylation at T308 is mediated by PDK-1 (3′-phosphoinositide-dependent kinase 1) while phosphorylation at S473 can be mediated by several kinases including PDK-1 and mTORC2 [13]. Once activated, Akt can phosphorylate many substrates to exert its functions. The best-characterized substrate of Akt is the serine/threonine kinase mTOR (mammalian target of rapamycin). Akt can directly phosphorylate and activate mTOR and indirectly activate mTOR by phosphorylating and inactivating tuberous sclerosis complex 2 (TSC2). Activated mTOR forms a complex with Raptor to activate ribosomal protein S6 kinases (S6K) and inhibit 4E-BP, leading to increased protein translation [13]. It is known that activation of the PI3K/Akt/mTOR pathway can increase cell survival and apoptosis resistance induced by a number of agents [12], [13]. For example, overexpression of Akt increases TRAIL resistance [11], [14]. On the other hand, the tumor suppressor gene PTEN (phosphatase and tensin homolog deleted on chromosome ten) can dephosphorylate PIP3 to function as a negative regulator of PI3K/Akt/mTOR signaling. PTEN inactivation leads to the activation of the PI3K/Akt/mTOR signaling pathway. However, the exact mechanism by which the PI3K/Akt/mTOR pathway confers TRAIL resistance is not fully understood.

In this study, we demonstrated that increased activation of the Akt pathway plays an important role in TRAIL resistance. Specifically, we showed that TRAIL activates the Akt pathway and that blockade of Akt activation by the PI3K inhibitor LY294002 or knockdown of Akt expression by siRNA sensitizes resistant cells to TRAIL. We also found that reintroduction of wild-type PTEN into PTEN knockout cells sensitizes PTEN^−/−^ cells to TRAIL whereas inactivation of PTEN in PTEN^+/+^ and PTEN^+/−^ cells leads to TRAIL resistance. These results suggest that increased activation of the Akt pathway via inactivation of PTEN plays an important role in TRAIL resistance.

## Materials and Methods

### Reagents

LY294002 was purchased from Alexis Biochemicals (San Diego, CA). Recombinant TRAIL was purchased from PeproTech (Rocky Hill, NJ). Rabbit polyclonal antibodies against phosphorylated Akt (Ser473), phosphorylated mTOR, phosphorylated p70S6K, phosphorylated eIF4E, PTEN, total Akt, and poly(ADP-ribose) polymerase (PARP) were purchased from Cell Signaling Technology (Beverly, MA). Anti-actin antibody was purchased from Sigma. The adenoviruses expressing wild-type and dominant negative PTEN, as well as control adenoviruses expressing galactosidase (Ad-LacZ), were kindly provided by Dr. Mengjer Lee (University of Louisville).

### Cell lines, culture conditions, and treatment

The human breast cancer T47D cells were obtained from American Type Culture Collection and maintained in DMEM with 10% fetal bovine serum (FBS). Human ovarian cancer SKOV3 cells were cultured in RPMI1640 with 10% FBS, OVCA432 cells were cultured in MCDB105/M199 medium with 10% FBS as described [15]. PTEN-knockout mouse prostate epithelial cells (PTEN^−/−^ and PTEN^+/−^) and normal cells (PTEN^+/+^) were obtained from Dr. Young Chen (Wake Forest University) and maintained in DMEM with 10% FBS, as described previously [16]. All cell lines were cultured at 37°C in a humidified atmosphere consisting of 5% CO_2_ and 95% air.

### siRNA transfection for knockdown of Akt

Signal Silence siRNA for Akt and corresponding control siRNA were purchased from Cell Signaling. The transfections were performed as suggested by the manufacturer with slight modifications, as described previously [17]. Briefly, T47D cells were plated at 6×10^5^ per well in six-well plates. The next day, cells were transfected with Akt or non-target control siRNAs using Oligofectamine (Invitrogen). After 24 h, transfected cells were trypsinized and plated at 6×10^5^ per well in six-well plates. 48 h later, transfected cells were left untreated or treated with TRAIL (100 ng/ml) for 24 h and then harvested for assessing the expression of Akt and cleaved PARP by Western blot analysis. To determine chemosensitivity, cells with or without transfection were placed at 8,000 per well in 96-well plates and then treated with TRAIL for 24 h, and cell viability was determined by 3-(4,5-dimethylthiazol-2-yl)-2,5-diphenyltetrazolium bromide (MTT) assays.

### MTT assays

MTT assays were described previously [18].

### Western blot analysis

The procedures for preparation of whole-cell protein lysates and Western blot analysis were described previously [18].

### Adenoviruse infections

PTEN^+/+^, PTEN^+/−^ and PTEN^−/−^ mouse prostate epithelial cells and human breast cancer T47D cells were plated in 60 mm dishes at 8×10^5^, 6×10^5^, 5×10^5^ and 8×10^5^ cells per dish, respectively. The next day, cells were washed with normal medium and infected with adenoviruses expressing wild-type PTEN, dominant negative PTEN, or LacZ. During infection, cells were gently shaken every 10 min for 1 h, and then maintained in adenovirus containing medium at 37°C in a humidified atmosphere containing 5% CO_2_. 24 h later, infected cells were switched to normal medium without adenoviruses and then continuously cultured for another 48 h. At 72 h after infection, cells were trypsinized and plated in 60 mm dish at 5×10^5^ cells. The next day, cells were left untreated or treated with TRAIL (100 ng/ml) for 48 h. Cells were harvested for Western blot analysis and the MTT assay, respectively.

### Statistical analysis

Statistical analysis was done using Student's *t* test. The data were presented as the mean ± SD, and *P*≤0.05 was considered significant.

## Results

### Characterization of TRAIL sensitivity in a panel of breast and ovarian cancer cell lines

**Figure 1:**
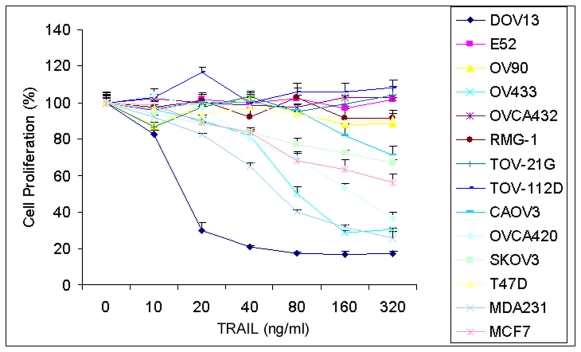
Effect of TRAIL on cell proliferation. Human breast cancer cell lines T47D, MDA231, and MCF7 and ovarian cancer cell lines DOV13, E52, OV90, OV433, OVCA432, RMG-1, TOV-21G, TOV-112D, CAOV3, OVCA420, and SKOV3 were treated with various doses of TRAIL for 24 h. Cell proliferation was determined by MTT assays. Cell proliferation data are expressed as percentage of untreated cells. Representative of three independent experiments.

To understand the mechanisms of TRAIL resistance, we first investigated TRAIL sensitivity in 14 cancer cell lines including 3 breast and 11 ovarian cancer cell lines. These cell lines were treated with different doses of TRAIL for 24 h, and cell proliferation was measured by the MTT assay. Fig. 1 shows that these cell lines exhibited the differential TRAIL sensitivity; MDA-231, DOV13, OV433 and OVCA420 cells were sensitive to TRAIL whereas the rest of lines were resistant to TRAIL except MCF-7 cells that showed modest resistance. These data show that many ovarian and breast cancer cell lines are resistant to TRAIL.

### The Akt/mTOR pathway is activated in TRAIL resistant cell lines

**Figure 2:**
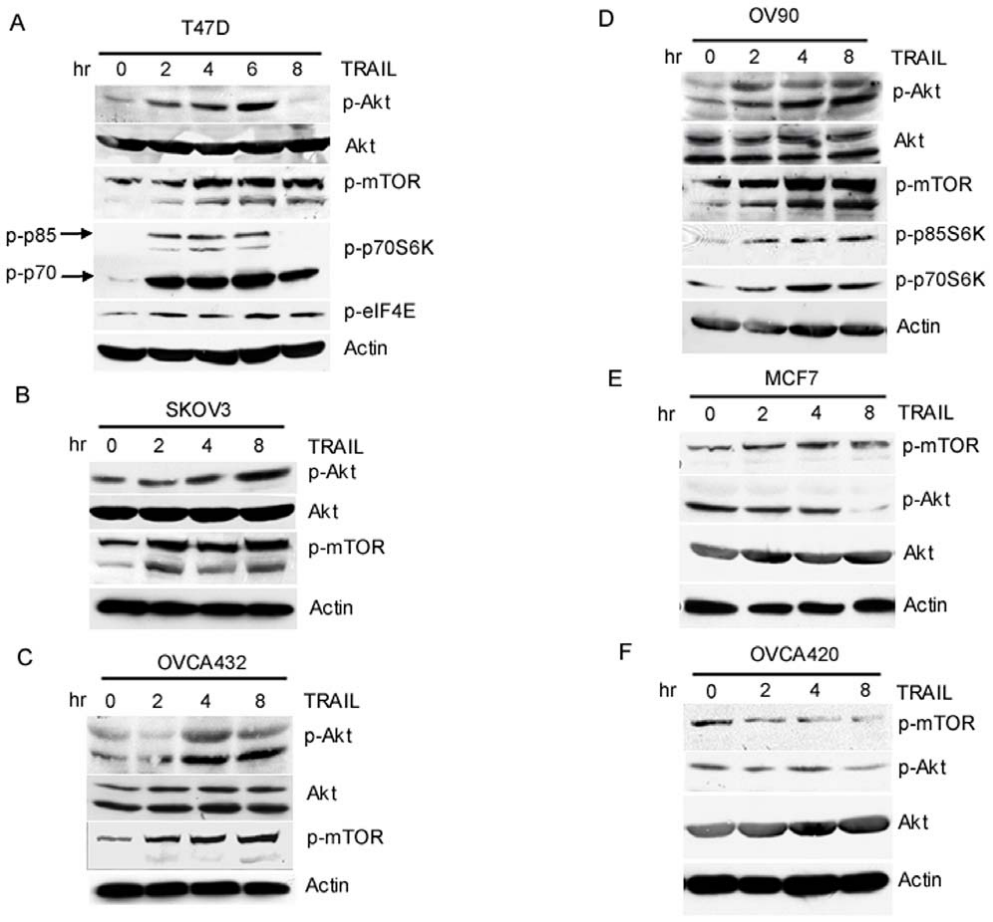
Activation of Akt/mTOR pathway by TRAIL in resistant cell lines but not in sensitive cell lines. T47D (A), SKOV3 (B), OVCA432 (C), OV90 (D), MCF7 (E) and OVCA420 (F) cells were treated with 100 ng/ml TRAIL for 0, 2, 4 and 8 h (in T47D cells, 6 h treatment was included), and total protein was extracted. The levels of total and phosphorylated Akt (*p-AKT*), and phosphorylated mTOR (*p-mTOR*) were determined by Western blot analysis. In T47D (A) and OV90 (D) cells, the levels of phosphorylated p-p70S6K and phosphorylated elF4E (p-elF4E) were also examined. β-actin was used as a loading control. Of note, there were two bands for Akt (Akt1 and Akt2) in both OVCA432 and OV90 cells while only one band for Akt was detected in T47D and SKOV3 cells.

Since many breast and ovarian cancer cell lines are resistant to TRAIL, it is important to understand the underlying mechanism of TRAIL resistance. It has been reported that TRAIL can activate several pro-survival pathways, including Akt, NF-κB and ERK pathways [19], [20], [21], [22]. Because activation of these pathways modulates cell survival and chemoresistance, it is conceivable that activation of these pathways contributes to TRAIL resistance. To test this possibility, we treated four resistant lines T47D, SKOV3, OVCA432 and OV90 with 100 ng/ml TRAIL for different periods of time and examined the activation of the Akt, NF-κB and ERK pathways, as well as expression of the Bcl-2 family proteins. As shown in Fig. 2A–D, the levels of both phosphorylated Akt and mTOR protein were increased as early as 2 h and lasted up to 6 h after TRAIL treatment, We also showed that such activation was not due to an increase in the total Akt and mTOR proteins, which remained constant (Fig. 2A–D). Moreover, we showed that p70S6K and elF4E, both are downstream targets of mTOR, are activated (Fig. 2A and D). These data indicate that TRAIL can activate the Akt/mTOR pathway in TRAIL resistant cell lines. Importantly, we showed that Akt and mTOR are not activated in TRAIL sensitive MCF-7 and OVCA420 cells (Fig. 2E and F). Thus, our results suggest that activation of Akt/mTOR pathway is a common event in TRAIL resistant cancer cell lines and that this pathway may be important in TRAIL resistance. In addition, we found that TRAIL could activate the MEK/ERK and NF-κB pathways in OV90 cells, but not in other three cell lines (data not shown). Furthermore, inhibition of the ERK and NF-κB pathways by their corresponding pharmacological inhibitors did not affect TRAIL sensitivity in OV90 cells (data not shown), indicating that the activation of these two pathways may not be important in TRAIL resistance in this cell line.

### Inhibition of Akt activation sensitizes resistant cells to TRAIL

**Figure 3:**
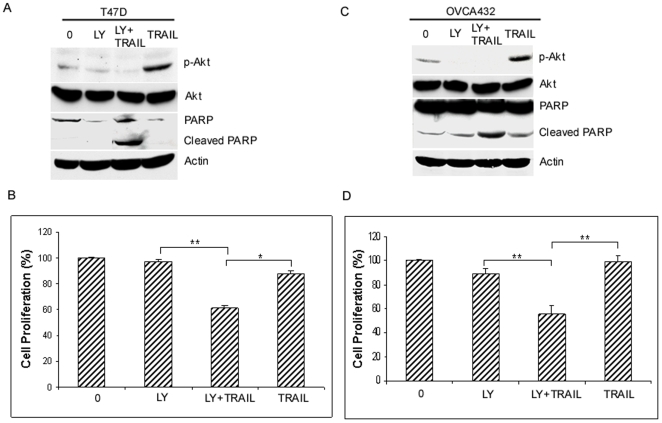
Inhibition of Akt activity sensitizes resistant cancer cells to TRAIL. A and C, effect of the PI3K inhibitor LY294002 on TRAIL-induced apoptosis. Breast cancer cell T47D (*A*) and ovarian cancer cell OVCA432 (*C*) were left untreated or pretreated with 10 µM LY294002 (*LY)* for 30 min, and then treated with or without 100 ng/ml TRAIL for 6 h. Total protein was extracted, and cleaved PARP and total and phosphorylated Akt were determined by Western blotting. *B and D*, effect of LY294002 on TRAIL-induced growth inhibition. T47D (*B*) and OVCA432 cells (*D*) were left untreated or pretreated with 10 µM LY294002 for 30 min, and then treated with or without 100 ng/ml TRAIL for 24 h. Cell proliferation was determined by MTT assays and expressed as percentage of untreated cells. Representative of three independent experiments. **, *P*<0.001, statistical significance; *, *P*<0.01, statistical significance.

Since the Akt pathway was activated in all resistant cell lines tested, we asked whether inhibition of Akt activity could increase their TRAIL sensitivity in resistant cells. To this end, we treated T47D cells with the PI3K inhibitor LY294002 and examined the effect of Akt inhibition on TRAIL-induced apoptosis. As shown in Fig. 3, TRAIL treatment increased Akt phosphorylation in T47D cells, which was abolished by LY294002, showing that Akt activation is PI3K dependent. Similarly, we also showed that activation of Akt is blocked by LY294002 in OVCA432 cells (Fig. 3C). Importantly, inhibition of Akt phosphorylation by LY294002 significantly increased TRAIL-induced PARP cleavage as compared to cells treated with TRAIL, or LY294002 alone (Fig. 3A and C). Furthermore, we showed that combined treatment of TRAIL and LY294002 significantly inhibits cell proliferation as compared to either agent alone (Fig. 3B and D). These results strongly suggest that the activation of Akt plays an important role in TRAIL resistance in cancer cells.

### Knockdown of Akt sensitizes breast cancer T47D cells to TRAIL

**Figure 4:**
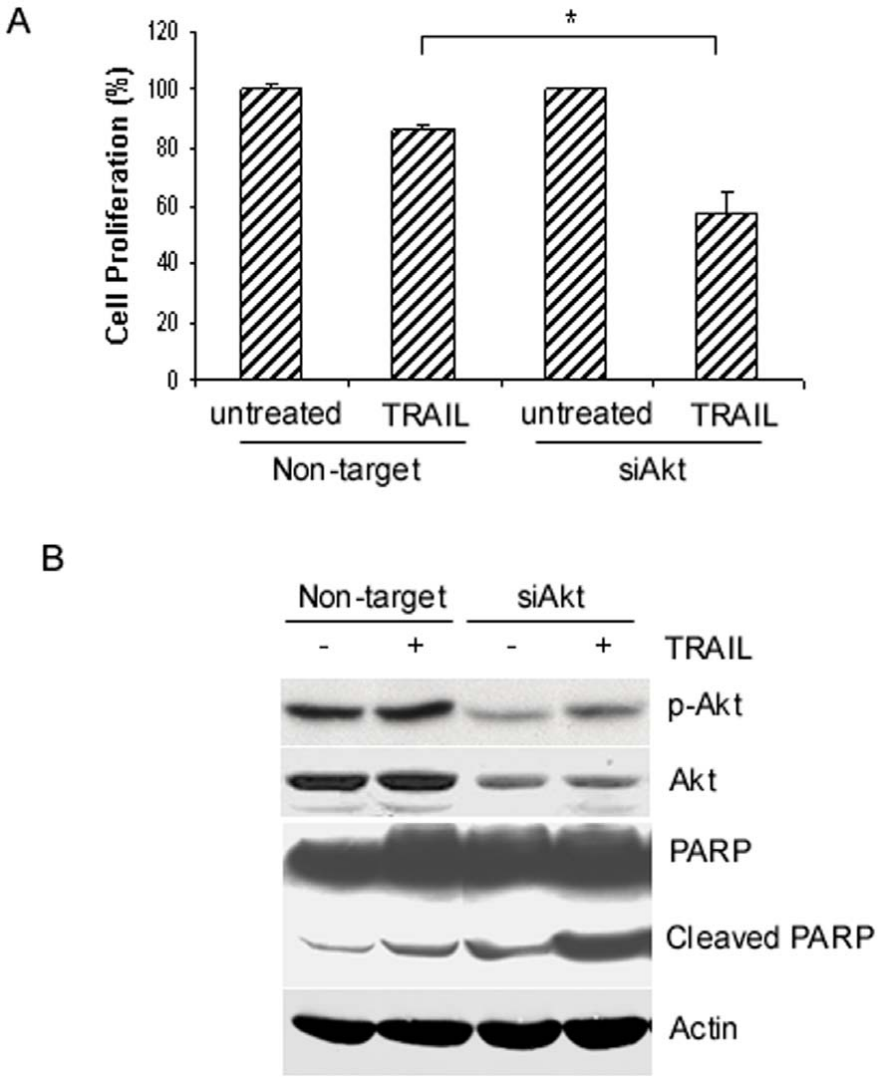
Knockdown of Akt sensitizes breast cancer T47D cells to TRAIL. *A*, effect of Akt knockdown on cell proliferation. T47D cells were plated at 6×10^5^ per well in six-well plates. The next day, cells were transfected with Akt or control siRNAs using Oligofectamine. After 2 d, cells were left untreated or treated with TRAIL (100 ng/ml) for 24 h, and cell proliferation was determined by MTT assays. Cell proliferation data are expressed as percentage of untreated cells. Representative of three independent experiments. *, *P*<0.01, statistical significance. *B*, effect of Akt knockdown on TRAIL-induced apoptosis. T47D cells were transfected with Akt or control siRNAs and then treated with or without TRAIL (100 ng/ml) for 24 h as described in (*A*). Total protein was extracted for assaying total and phosphorylated Akt (*p-AKT*) and PARP by Western blot analysis. β-actin was used as a loading control.

Although treatment with the PI3K inhibitor LY294002 increased TRAIL-induced apoptosis, the results may not completely reflect the exclusive role of Akt in TRAIL resistance because LY294002 may inhibit other kinases. To investigate the direct role of Akt activation in TRAIL resistance, we used siRNA to knockdown Akt expression and then determine the effect of Akt knockdown on TRAIL sensitivity. We transfected T47D cells with siRNA against Akt (Akt 1, 2 and 3) or control siRNA and showed that Akt was efficiently knock downed by Akt siRNA in cells with or without TRAIL treatment, as compared to cells transfected with control siRNA (Fig. 4B). Importantly, we showed that knockdown of Akt sensitized TRAIL induced PARP cleavage as compared to the same cells transfected with control siRNA (Fig. 4B). Further, knockdown of Akt increased TRAIL-induced growth inhibition while such changes were minimal in cells transfected with control siRNA (Fig. 4A). Thus, our results indicate that while TRAIL can cause apoptosis, it also activates the Akt survival pathway to counteract TRAIL-induced apoptosis.

### Loss of PTEN confers TRAIL resistance

**Figure 5:**
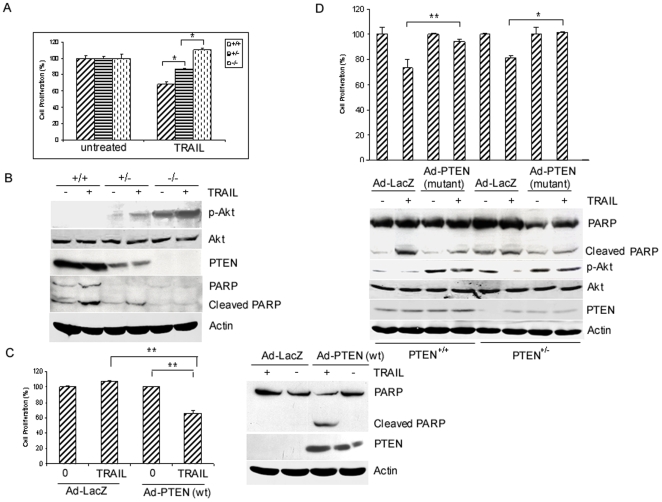
The role of PTEN in TRAIL sensitivity. *A*, effect of loss of PTEN in TRAIL-induced growth inhibition. Mouse prostate epithelial cells with wild type PTEN (PTEN^+/+^), PTEN knockout (PTEN*^+/−^*) and heterozygous (PTEN*^−/−^*) were plated in 96-well plates, and then treated with 100 ng/ml TRAIL for 48 h. Cells proliferation was determined by MTT assays and expressed as percentage of untreated cells. Representative of three independent experiments. *, *P*<0.01, statistical significance. *B*, effect of PTEN loss on TRAIL-induced apoptosis. PTEN^+/+^, PTEN^+/−^, and PTEN^−/−^ cells were treated with 100 ng/ml TRAIL for 48 h as described in (*A*). Total protein was extracted for assaying total and phosphorylated Akt (*p-AKT*), PTEN and PARP by Western blot analysis. β-actin was used as a loading control. *C*, restored PTEN expression sensitizes PTEN^−/−^ cells to TRAIL. PTEN^−/−^ cells (5×10^5^) were infected with adenoviruses expressing wild-type PTEN (*Ad-PTEN wt*) or expressing LacZ (*Ad-lacZ*). At 72 h after infection, cells were trypsinized and plated in 60 mm dish at 5×10^5^ cells. The next day, cells were left untreated or treated with TRAIL (100 ng/ml) for 48 h. Cells were harvested for MTT assays (*Upper panel*) and Western blot analysis was employed for detecting the levels of PARP and PTEN proteins (*lower panel*), respectively. Cell proliferation data were expressed as percentage of untreated cells. Representative of three independent experiments. **, *P*<0.001, statistical significance. *D,* inhibition of PTEN function by a dominant negative form of PTEN confers TRAIL resistance. PTEN^+/+^ and PTEN^+/−^ cells were infected with adenoviruses expressing a dominant negative form of PTEN for 72 h and then treated with TRAIL (100 ng/ml) for 48 h. Cells were either used for growth inhibition by MTT assays (*upper panel*) or harvested for detecting the levels of PARP, PTEN, and total and phosphorylated Akt by Western blot analysis (lower panel). Cell proliferation data were expressed as percentage of untreated cells. Representative of three independent experiments. *, *P*<0.01, statistical significance. **, *P*<0.001, statistical significance.

It has been known that PTEN is a negative regulator of Akt and that increased Akt activity has been associated with inactivation of PTEN in several cancers. To understand the mechanism of Akt activation-mediated TRAIL resistance, we treated PTEN^+/+^, PTEN^+/−^ and PTEN^−/−^ mouse prostate epithelial cells with TRAIL for 48 h and measured cell proliferation by MTT assays. Fig. 5A shows that TRAIL-induced about 32% growth inhibition in PTEN^+/+^ cells whereas TRAIL did not affect cell proliferation in PTEN^−/−^ cells, while PTEN^+/−^ cells showed about 13% growth inhibition after TRAIL treatment. Furthermore, we showed that Akt is activated in PTEN^−/−^ cells but not in PTEN^+/+^, while minimal activation in PTEN^+/−^ cells. Importantly, TRAIL treatment caused significant PARP cleavage in PTEN^+/+^ cells, moderate cleavage in PTEN^+/−^ cells and complete absence of cleavage in PTEN^−/−^ cells (Fig. 5B). These results suggest that PTEN is required for TRAIL-induced apoptosis in mouse prostate epithelial cells.

Since PTEN^−/−^ cells are resistant to TRAIL, we asked whether restoration of PTEN expression affects TRAIL sensitivity in PTEN^−/−^ cells. PTEN^−/−^ cells were infected with adenoviruses expressing wild-type PTEN or LacZ. As shown in Fig. 5C, PTEN expression was restored in cells infected with adenoviruses expressing PTEN but not in cells infected with adenoviruses expressing LacZ. Importantly, we were able to show that restoration of PTEN expression renders PTEN^−/−^ cells sensitive to TRAIL induced apoptosis and growth inhibition whereas such effects were not observed in cells infected with control viruses (Fig. 5C). Since PTEN^+/+^ cells are sensitive to TRAIL, we asked whether inhibition of PTEN function affects TRAIL-induced apoptosis in PTEN^+/+^ cells. To this end, we infected PTEN^+/+^ and PTEN^+/−^ cells with adenoviruses expressing a dominant negative form of PTEN or LacZ, and treated infected cells with TRAIL for 48 h. Fig. 5D shows that introduction of dominant negative form of PTEN increased p-Akt level in PTEN^+/+^ cells. Importantly, introduction of a dominative negative form of PTEN renders PTEN^+/+^ and PTEN^+/−^ cells more resistant to TRAIL (Fig. 5D). Consistent with the role of Akt in inhibiting apoptosis, TRAIL induced PARP cleavage was decreased dramatically in PTEN^+/+^ and PTEN^+/−^ cells after they were infected with viruses expressing a dominant negative form of PTEN. Furthermore, we showed that TRAIL-induced growth inhibition in PTEN^+/+^ cells infected with an adenovirus expressing a dominant negative form of PTEN was about 5% but about 27% in the same cells infected with control adenoviruses (Fig. 5D). We also showed that in PTEN^+/−^ cells infected with dominant negative form of PTEN, cell proliferation was about 102% as compared to the same cells infected with control viruses in which cell proliferation was about 81% upon TRAIL treatment (Fig. 5D, upper panel).

### The role of PTEN in TRAIL sensitivity in breast cancer T47D cells

**Figure 6:**
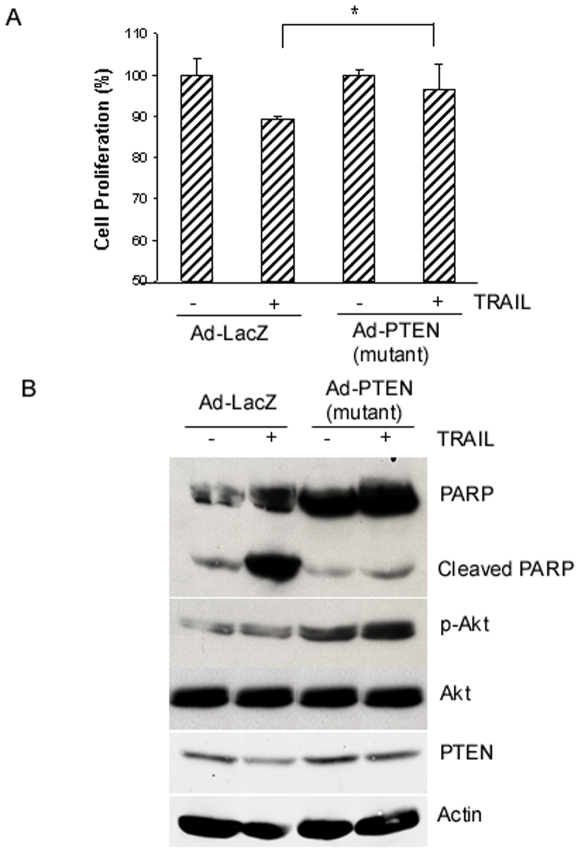
Inhibition of PTEN function renders breast cancer T47D cells more resistant to TRAIL. *A*, effect of inhibition of PTEN function on cell proliferation. T47D cells (8×10^5^) were infected with adenovirus expressing a dominant negative form of PTEN or adenoviruses expressing LacZ for 72 h and then treated with TRAIL (100 ng/ml) for 48 h. Cell proliferation was determined by MTT assays. Cell proliferation data are expressed as percentage of untreated cells. Representative of three independent experiments. *, *P*<0.01, statistical significance. *B*, effect of inhibition of PTEN function on apoptosis. T47D cells infected with adenoviruses as described in A were treated with TRAIL (100 ng/ml) for 48 h and then harvested for analyzing the levels of PARP, PTEN, total and phosphorylated Akt proteins by Western blot analysis (*lower panel*). β-actin was used as a loading control.

Since breast cancer T47D cells with a wild-type PTEN were resistant to TRAIL (Fig. 1), we asked whether modulation of PTEN function would affect TRAIL sensitivity in T47D cells. To this end, we infected T47D cells with adenoviruses expressing a dominant negative form of PTEN or control viruses and then treated infected cells with TRAIL (100 ng/ml) for 48 h, and the effect of such treatment on TRAIL sensitivity was determined. Fig. 6 shows that cells infected with adenovirus expressing a dominant negative form of PTEN express a higher level of p-Akt while such an increase was not observed in cells infected with adenoviruses expressing LacZ, which suggests that PTEN function was inhibited by a dominant negative form of PTEN (Fig. 6B). Consistent with the role of Akt in inhibiting apoptosis, cleaved PARP was significantly reduced in cells expressing a dominant negative form of PTEN as compared to cells expressing control viruses (Fig. 6B). Furthermore, overexpression of a dominant negative form of PTEN renders T47D cells more resistant to TRAIL as compared to the same cells infected with viruses expressing LacZ (Fig. 6). Therefore, these data suggest that increased Akt activation due to inactivation of PTEN could confer TRAIL resistance in cancer cells.

## Discussion

In this study, we show that TRAIL activates the Akt survival pathway in TRAIL-resistant cancer cell lines. We also show that the underlying mechanism may be due to loss of functional PTEN because PTEN knockout mouse prostate epithelial cells are resistant to TRAIL while cells with intact PTEN are sensitive to TRAIL. Restoring PTEN expression rendered PTEN^−/−^ cells sensitive to TRAIL. Further, inactivation of PTEN enhances the level of TRAIL resistance in T47D cells. These findings suggest that activation of the Akt survival pathway plays a critical role in TRAIL resistance and that the Akt pathway is a potential therapeutic target to enhance TRAIL-based therapy.

The activation of the Akt pathway has been implicated in protecting cells by many death stimuli, thereby conferring cell survival [10], [13]. Previous studies suggested that overexpression of Akt and its upstream regulator PI3K increased TRAIL resistance in breast and ovarian cancer cells [23], [24]. However, the underlying mechanism of its resistance is not fully understood. In this study, we showed that while TRAIL induces apoptosis in cancer cells, it also activates several survival pathways, which may counteract TRAIL-induced apoptosis, leading to resistance. In many TRAIL resistant breast and ovarian cancer cell lines but not sensitive cell lines, the Akt pathway was activated (Fig. 2), suggesting that the activation of the Akt pathway may be a common event in TRAIL resistant cells. Consistent with this notion, we found that inhibition of Akt activation by its pharmacological inhibitor LY294002 or knockdown of its expression by siRNA sensitizes TRAIL-resistant cells to TRAIL. Collectively, these data strongly suggest that activation of the Akt survival pathway plays a critical role in TRAIL resistance in ovarian and breast cancer cells and imply that inhibition of this survival pathway might overcome TRAIL resistance.

We also showed that inhibition of PI3K activity blocked Akt activation in TRAIL resistant cell lines. Since PI3K can serve as an upstream positive regulator of Akt, these results suggest that increased Akt activation is at least in part through the increased activation of PI3K activity. At the downstream level, it is well known that Akt exerts its biological functions by phosphorylating its downstream substrates including mTOR. Activation of mTOR can increase protein translation, resulting in cell survival [13]. Consistent with this, we found that TRAIL treatment can cause mTOR phosphorylation and subsequent activation. Our data suggest that TRAIL may activate the PI3K/Akt/mTOR pathway to counteract TRAIL-induced apoptosis, leading to TRAIL resistance.

In cancer cells, the Akt survival pathway can be activated by the several mechanisms including inactivation of its upstream negative regulator PTEN. By dephosphorylating and conversing PIP3 to PIP2, PTEN inhibits Akt activation and subsequently sensitizes cells to death [10], [13]. Consistent with the role of the PI3K/Akt pathway in TRAIL resistance, we found that loss of PTEN in mouse prostate epithelial cells confers TRAIL resistance whereas the same cells with intact PTEN are sensitive to TRAIL while PTEN^+/−^ cells show intermediate resistance. These data suggest that PTEN indeed plays an important role in TRAIL sensitivity. Furthermore, we showed that reintroduction of a mutant form of PTEN into TRAIL resistant T47D cells further increases TRAIL resistance. Collectively, these data indicate that in TRAIL resistant cells, loss of PTEN contributes to TRAIL resistance. It has been known that decreased or loss of PTEN expression occurs in at least 50% of breast and ovarian cancers [25]. It is possible that TRAIL resistant breast and ovarian cancer cells have decreased levels of PTEN, which need further investigation. Further, a recent study showed that overexpression of miR-221&222 down regulates PTEN, leading to increased activation of Akt and subsequent TRAIL resistance [26]. Additionally, TRAIL resistant leukemia cells expressed a higher level of phosphorylated inactive form of PTEN, which resulted in an increase in Akt activation and TRAIL resistance [27]. In addition to the role of PTEN in regulating Akt activation, several studies have shown that Akt activation by TRAIL treatment can be due to the other mechanisms. For example, TRAIL was shown to activate Src, leading to Akt activation and TRAIL resistance [28]. Another mechanism involves DNA-PKcs-mediated Akt activation [29]. Nevertheless, the mechanisms by which TRAIL activates Akt are still not fully understood.

Although TRAIL selectively kills cancer cells but not normal cells, many cancer cells including breast and ovarian cancer cells are resistant to TRAIL [30], [31]. Accumulating evidence indicates that TRAIL resistance involves many molecules and pathways including PI3K/Akt pathway [4]. Although previous studies showed that overexpression or constitutive expression of Akt confers TRAIL resistance in cancer cells [11], [14], this study is unique because our work indicates that inducible activation of the Akt/mTOR survival pathway counteracts TRAIL-induced apoptosis, leading to TRAIL resistance, as opposed to previous studies in which overexpression or constitutive activation of Akt caused TRAIL resistance [11], [14]. Our conclusion is based on the fact that Akt and its downstream target mTOR are activated in all TRAIL resistant cell lines tested. Further, we were able to increase TRAIL sensitivity by inhibiting the Akt pathway using its pharmacological inhibitor LY294002 or siRNA against Akt.

In summary, we demonstrate that TRAIL activates the Akt survival pathway. We also demonstrate that inhibition of the Akt survival pathway sensitizes breast and ovarian cancer cells to TRAIL. More importantly, we demonstrate that loss of PTEN confers TRAIL resistance in both mouse prostate epithelial and human breast T47D cells. Therefore, our findings suggest that targeting the Akt survival pathway could overcome TRAIL resistance in breast and ovarian cancer cells.
